# Systematic review and meta-analysis of the relationships between real-time neurofeedback training parameters and acquisition of neural modulation

**DOI:** 10.3389/fnhum.2025.1652607

**Published:** 2025-08-29

**Authors:** Eldrick V. Galang, Miguel A. Velásquez, Dehan Elcin, Samantha O'Connell, Julian Wieck, Savannah McNair, Paul J. Colombo

**Affiliations:** ^1^Department of Cognitive Science, School of Social Sciences, University of California, San Diego, San Diego, CA, United States; ^2^Department of Psychology, School of Science and Engineering, Tulane University, New Orleans, LA, United States; ^3^Department of Epidemiology, School of Public Health and Tropical Medicine, Tulane University, New Orleans, LA, United States; ^4^School of Human Sciences and Humanities, Department of Psychological and Behavioral Health Sciences, University of Houston – Clear Lake, Houston, TX, United States; ^5^Department of Human Development and Quantitative Methodology, College of Education, University of Maryland, College Park, MD, United States; ^6^Brain Institute, Tulane University, New Orleans, LA, United States

**Keywords:** neurofeedback, brain-computer interface, cognitive control, biofeedback, neural plasticity, meta-analysis

## Abstract

**Introduction:**

Real-time neurofeedback is an emerging method for regional modulation of neural activity with physiological and behavioral effects that persist beyond the duration of feedback sessions. However, many individuals fail to achieve successful modulation, a challenge known as the “neurofeedback inefficacy problem.” This study examined how methodological parameters of neurofeedback training influence the acquisition and retention of neural modulation in healthy adults.

**Methods:**

A systematic search identified eligible studies, resulting in 55 participant groups included in the meta-analysis. Standardized mean differences (Hedges' g) were calculated for changes in neural activity from first to last session and from pre- to post-training. Subgroup analyses and meta-regression were conducted to assess the impact of discrete and continuous moderators.

**Results:**

This meta-analysis identified four parameters associated with significant neural modulation in the desired direction: the neurofeedback imaging device used, complexity of the feedback stimulus, presence of a pre-training rehearsal trial, and EEG target oscillations.

**Discussion:**

This meta-analysis highlights key methodological factors that shape neurofeedback efficacy in non-clinical populations. Findings may serve to better understand how methodological variables used in neurofeedback influence the acquisition and retention of neural modulation.

**Systematic review registration:**

https://www.crd.york.ac.uk/PROSPERO/view/CRD42022357160, identifier: CRD42022357160.

## 1 Introduction

Real-time neurofeedback is a type of biofeedback that offers a non-invasive method by which to self-regulate neural activity. By continuously monitoring an individual's current brain state and delivering stimuli when self-modulation aligns with predefined target states, neurofeedback systems typically implement reinforcement-based learning, in which individuals receive contingent feedback to shape their neural activity (Sitaram et al., 2017). While not all neurofeedback relies on reinforcement mechanisms—some studies explore endogenous neuromodulation without external reinforcement feedback (Othmer, 2023)—the present study focuses specifically on reinforcement learning-based neurofeedback. Prior work suggests that physiological and behavioral effects of reinforcement-based neurofeedback training persist beyond the duration of feedback sessions (Rance et al., 2018; Van Doren et al., 2019). This process facilitates neural adaptations by strengthening pathways associated with optimal cognitive or affective functioning (Loriette et al., 2021).

The non-invasive nature of neurofeedback training makes it an attractive alternative to pharmacological intervention and other invasive methods, particularly in clinical settings. Neurofeedback has been shown to improve hyperactivity regulation in attention-deficit hyperactivity disorder (ADHD) (Enriquez-Geppert et al., 2019), as well as alleviate symptoms in major depressive disorder (MDD) (Young et al., 2014) and post-traumatic stress disorder (PTSD) (Nicholson et al., 2017). In addition, it has demonstrated effectiveness in epilepsy by reducing dysregulated brainwave patterns (Sterman and Egner, 2006) and has aided stroke rehabilitation patients in regaining motor control (Cervera et al., 2018). Neurofeedback has also expanded into non-clinical applications, increasingly being utilized in athletics and the creative arts to manage stress and optimize performance (Mirifar et al., 2017; Gruzelier, 2014). Moreover, neurofeedback has been employed to facilitate relaxation and concentration within mindfulness interventions by modulating the default mode network (Garrison et al., 2013), as well as improving emotion regulation through the down-regulation of amygdala activity (Herwig et al., 2019).

Of interest, approximately 38% of participants have been reported to be unsuccessful in using neurofeedback to modulate neural activity, a phenomenon referred to as the “neurofeedback inefficacy problem” (Alkoby et al., 2018). This issue may arise due to various factors, including individual differences in baseline neural activity (Hellrung et al., 2022), challenges in maintaining engagement and motivation during training (Kadosh and Graham, 2019), or psychological factors such as cognitive load (Bauer and Gharabaghi, 2015). Efforts have been made to standardize reporting across neurofeedback literature to enhance reproducibility and transparency in study findings. Ros et al. (2020) presented a “consensus on the reporting and experimental design of clinical and cognitive-behavioral neurofeedback studies” (CRED-NF) checklist of reporting standards to isolate the mechanisms that may result in changes in neurofeedback training outcomes. Despite these efforts, a critical gap remains in our knowledge regarding which specific methodological parameters are most influential in determining neurofeedback success. The present meta-analysis addresses this gap by systematically investigating how various training parameters affect neurofeedback-mediated neural modulation outcomes.

The current study focuses on how methodological variables used in neurofeedback influence the acquisition and retention of neural modulation. That is, given the variability in experimental protocols between neurofeedback interventions, the current study investigates the parameters that lead to the greatest neural change effect. For the purposes of this meta-analysis, acquisition is defined as a measured change in the ability to modulate neural activity using neurofeedback within a training session, and retention is defined as the sustained improvement in the ability to modulate neural activity between sessions or after a delay. To avoid the confounding effects of variability attributed to clinical disorders, the current study focuses solely on non-clinical populations. Clinical populations may be more susceptible to factors unrelated to biofeedback, as described by Arns et al. (2014), in which those with inattention and hyperactivity were more impacted by patient-therapist interactions rather than the feedback itself. It was hypothesized that longer training durations, presence of explicitly instructed strategies, use of functional localizers, and pre-training rehearsals would produce the largest effect sizes in neural modulation outcomes.

Previous systematic reviews and meta-analyses have studied neurofeedback success criteria and training parameters in clinical populations (Haugg et al., 2021; Fede et al., 2020; Fernández-Alvarez et al., 2022; Rahmani et al., 2022); however, the current meta-analysis is the first to examine training parameters in relation to direct outcome measures by assessing quantitative changes in neural signals among healthy individuals and to identify relationships between training parameters, acquisition, and retention of successful neurofeedback-mediated neural modulation. To determine parameters to be extracted for statistical evaluation, we used common experimental variables for neurofeedback studies—including those mentioned in the CRED-NF checklist—as well as parameters already reported in isolation in other systematic reviews, such as the presence of pre-training rehearsal and functional localizers (Haugg et al., 2020), extrinsic motivators such as monetary rewards (Kleih and Kübler, 2013), and presence of explicitly instructed strategies (Marxen et al., 2016). These parameters were compared using subgroup analysis, with effect sizes representing the standardized mean differences that reflect changes in neural activity elicited by neurofeedback training. This approach allowed us to examine how training-related experimental variables influence the acquisition and retention of voluntary neural modulation using neurofeedback in neurotypical adults.

## 2 Materials and methods

### 2.1 Literature search and study selection strategy

The systematic review and meta-analysis were conducted and reported in accordance with the Cochrane Handbook for Systematic Review of Intervention guidelines (Higgins et al., 2022) and the Preferred Reporting Items for Systematic Reviews and Meta-Analyses (PRISMA) (Page et al., 2021). The experimental protocol was pre-registered with the International Prospective Register of Systematic Reviews (PROSPERO) database (registration number: CRD42022357160).

To identify all research articles that contained neurofeedback interventions with healthy subjects, a comprehensive systematic literature search of three online databases was performed: PubMed, Embase, and Web of Science. The search included studies published through August 2022, with abstraction procedures conducted from August 2022 to January 2024. The terms used to identify and broadly capture all studies related to neurofeedback, included these: neurofeedback, brainwave biofeedback, EEG feedback, electromyography feedback, electroencephalographic biofeedback, and hemoencephalography.

### 2.2 Eligibility criteria

Studies not published in English, case-reports, reviews, commentaries, letters to the editor, meta-analyses, dissertations, and conference abstracts were excluded. The inclusion criteria for research article selection was as follows: (1) the study population included participants at least 18 years of age from a non-clinical population; (2) the study design consisted of neurofeedback interventions that included feedback of brain activity presented in near real-time to the participant; (3) the intervention made use of one of the following neuroimaging methods: functional magnetic resonance imaging (fMRI), functional near-infrared spectroscopy (fNIRS), electroencephalography (EEG), or magnetoencephalography (MEG); (4) Studies reported an outcome measure reflecting a change as a result of training as it relates to acquisition or retention values; (5) A measure of variance, or information to derive variance, was reported; (6) The study was a reviewed empirical study.

### 2.3 Data extraction

Five investigators (EG, MV, DE, SM, JW) extracted data from articles generated by the previously established literature search strategy. Combinations of two investigators screened and extracted the articles, with a third investigator resolving discrepancies for consensus. All articles identified through the keyword search from the online databases were migrated to Covidence for screening firstly at the level of their titles and abstracts, followed by a second screening using the full text of the studies. Those that met eligibility criteria at this stage (*N* = 39) were included in the meta-analysis, at which point meta-data and parameter details were extracted.

### 2.4 Neurofeedback parameters

A fully annotated list of extracted parameters and their categorizations to be compared to change in neurofeedback control can be found in the data abstraction form in the Supplementary material. Briefly, categorical parameters extracted included: study blinding, neuroimaging device used, participant characteristics (e.g., sex), strategies used for control of neurofeedback stimulus, the presence of explicit instructions given to develop control, presence of extrinsic motivation, neurofeedback display details, neurofeedback stimulus presentation timing, details of task demands between neurofeedback training intervals, signal modulation directionality, frequencies targeted, regions of interests targeted (cortical frontal, central, posterior), presence of pre-training rehearsal, presence of functional localizers.

Continuous parameters included details such as: participant characteristics (e.g., sample size, age), duration of neurofeedback training trials, intertrial interval, trials per block, interblock interval, blocks per session, intersession interval, number of sessions, duration of sessions, total neurofeedback training duration, total time spent in an experimental setting.

Neurofeedback display categories were defined as the following: simple audio refers to feedback that uses audio alone without visual components; simple visual includes stimuli that consist only of visual elements that are minimally animated, such as bars or line graphs; simple audiovisual uses stimuli that combine both minimal audio and visual components (e.g., a bar graph paired with a tone); complex displays involve stimuli that include multiple elements working together in a dynamic and interactive way (e.g., animations, games, or dynamically changing shapes).

### 2.5 Statistical analysis

#### 2.5.1 Effect sizes

Outcome measures from individual studies (most commonly EEG amplitude (μV) or percent signal change in BOLD fMRI) were converted to standardized effect sizes using the standardized mean difference. Due to the wide variability in how studies reported their outcome measures, the meta-analysis included two types of standardized mean differences: (1) post-training values minus pre-training baseline values, and (2) the final training session values minus the first training session values. All analyses were performed separately for these two groups. The within-group standardized mean differences (SMD) were calculated by subtracting the final value from the initial value and dividing it by the pooled standard deviation: $SMD=\;\frac{\chi_{t2}-\chi_{t1}}{SD_{Pooled}}$,

where *SD*_*Pooled*_ is the sum of the two squared standard deviations divided by two (Harrer et al., 2021). The standard errors (SE) of within-group standardized mean differences were computed using the following formula:

$SE_{SMD}=\;\sqrt{\frac{2\left(1-r_{t1t2}\right)}{n}+\frac{SMD^{2}}{2n}}$ (Harrer et al., 2021; Borenstein et al., 2011, chap. 4; Becker, 1988).

As the correlation between initial and final values were unknown, 0.8 was used to impute *r* (Gnambs and Schroeders, 2024). Importantly, due to the small sample sizes in the studies (*Mean N* = 16.23, *SD* = 12.67) the Hedges' *g* correction was applied to all SMD values (Hedges, 1981). Hedges' *g* was used as the main effect size measure for all subsequent analyses.

To adjust for the direction of effect, the SMD was multiplied by −1 in studies where the aim of the neurofeedback task was to decrease activity, to ensure that a positive SMD indicated a change in the predicted direction.

#### 2.5.2 Influential case detection

Due to high heterogeneity between studies (*I*^2^ > 50%) an influence analysis was performed by leave-one-out analysis to determine how much the pooled effect sizes and heterogeneity measures were influenced by each study individually. Studies were classified as outliers if they had effect sizes that were lower than quartile 1 minus 1.5 times the interquartile range or greater than quartile 3 plus 1.5 times the interquartile range. Sensitivity analyses were conducted to determine the impact of influential cases on pooled effect sizes.

### 2.6 Meta-analysis

A random-effects model was used to pool effect sizes given considerable heterogeneity between studies. The Restricted Maximum Likelihood (REML) method was used for estimating the between study variance, as it has been reported to result in reduced bias (Veroniki et al., 2016). Knapp-Hartung adjustments (Knapp and Hartung, 2003) were used to calculate the confidence interval around the pooled effect (Harrer et al., 2021). Effects were weighted using their inverse variance to account for their precision. Heterogeneity across studies was assessed using the *Q* and *I*^2^statistics.

Subgroup analyses were conducted to investigate the effect of discrete moderators on the SMDs while meta-regression was used for continuous moderators. Predictors with too few cases per level (< 2) were excluded from the analysis. Continuous data with skewed distributions were log-transformed before analysis. The R package meta was used for pooled effect size, subgroup and meta-regression analyses (Balduzzi et al., 2019).

### 2.7 Quality assessment and bias

The same multi-investigator resolution protocol was used with the NIH Quality Assessment Tool for Before-After (Pre-Post) Studies with No Control Group (National Heart Lung Blood Institute, 2014) to examine the quality of the selected studies. The following quality criteria were assessed: the formalization of the research question, eligibility criteria and study population, the study participants' representativeness of clinical populations of interest, consistency in enrolled participants with the research question, sample size, clarity of intervention method, the clarity and validity of outcome measures, blinding, follow-up, statistical analysis, the frequency of measurement of outcomes, and group-level intervention details. Reviewers could select “yes,” “no,” or “cannot determine/not reported/not applicable” in response to each item in the tool. Each study was given a score ranging from 1–12 with categorical thresholds labeling them as either fair (8–10), or good (10–12) to add as an additional parameter by which to assess neurofeedback training reporting. If the studies failed to meet 5 or more quality criteria, they were reviewed by the team to determine whether they posed significant risk of bias and were rated as “poor” if so. The possibility of publication bias was assessed through visual inspection of funnel plots and by using Egger's regression test (Egger et al., 1997) to detect funnel plot asymmetry.

## 3 Results

### 3.1 Research Study Selection

**Figure 1:**
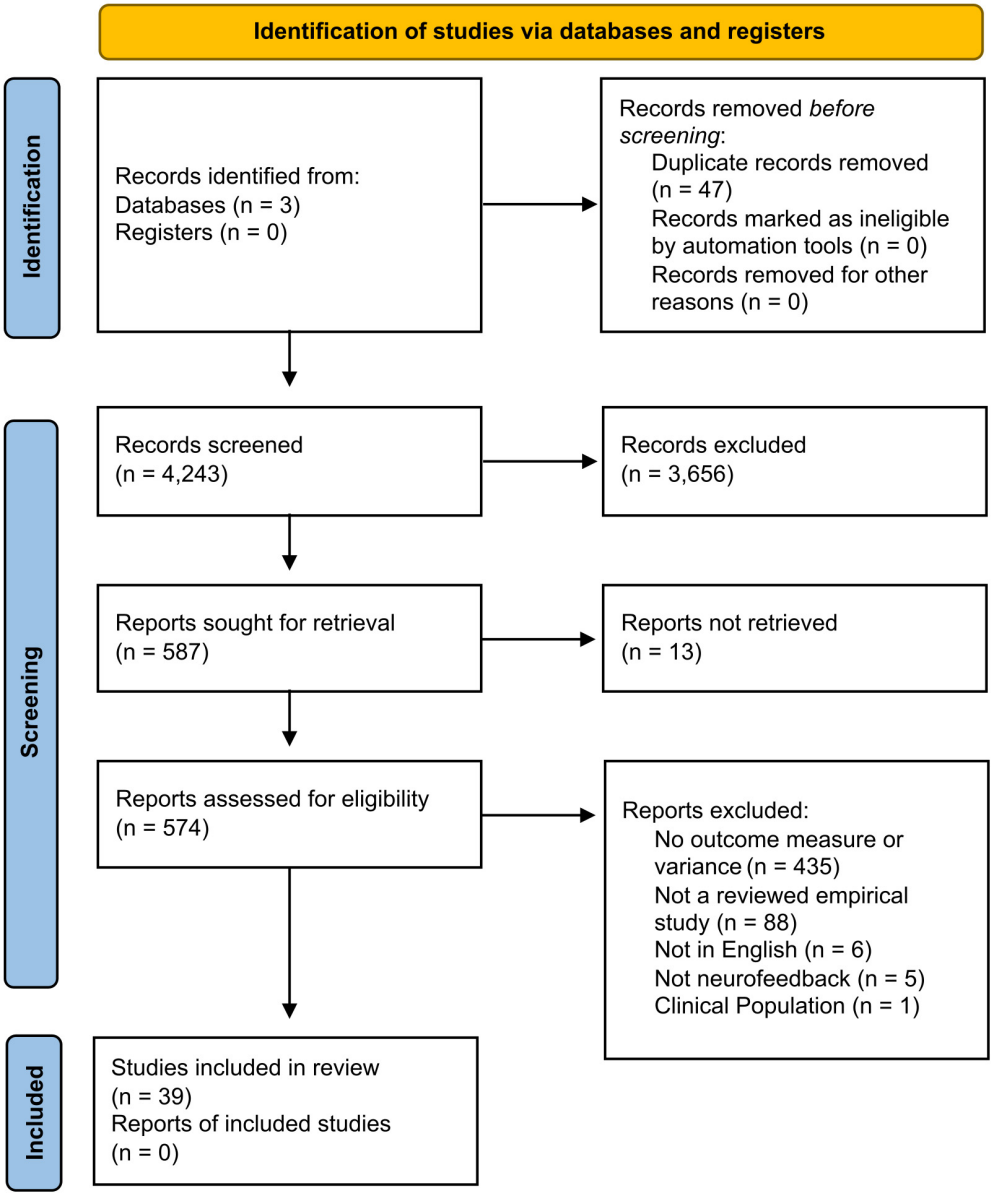
Neurofeedback intervention parameterization flow diagram of the inclusion criteria of studies eligible for meta-analysis. Flow diagram template adopted from the PRISMA approach to meta-analysis (Page et al., 2021).

Search keywords across three databases returned 4,243 unique articles for screening. After applying inclusion and exclusion criteria to title, abstract, and full-text screening, a total of 39 articles were found eligible for meta-analysis (Figure 1). Among these articles a total of 55 eligible participant groups were included in the analysis as separate data points. A full table with study characteristics, meta-data, and key parameters can be found in the Supplementary material.

### 3.2 Study characteristics

A full table with study characteristics and meta-data can be found in the Supplementary material.

### 3.3 Quality assessment and assessment of risk of bias

Quality assessment through the NIH Tool for Before-After (Pre-Post) Studies with No Control Group determined that there were 11 studies that were rated to have low risk of bias (“good”) while 34 were susceptible to some bias (“fair”). After careful evaluation, none of the selected studies were considered to have significant risk of bias (“poor”), therefore no studies were excluded based on the quality assessment. Studies and their scores regarding the quality assessment criteria can be found in the Supplementary material.

### 3.4 Results

Analysis of change in neural activation from first to last training sessions showed a significant effect of neurofeedback training (*SMD* = 0.32, *95% CI* 0.19 to 0.44, *p* < 0.001, *I*^2^ = 76.0%). Leave-one-out analysis detected one influential outlier (van Son et al., 2020: Group B: 8 Sessions of training). After removing this influential case there was a small increase in the pooled effect size (*SMD* = 0.34, *95% CI* 0.23 to 0.44, *p* < 0.001, *I*^2^ = 71.8%). Analysis of change in neural activation from pre-training baseline to post-training also resulted in a significant effect of training (*SMD* = 0.26, *95% CI* 0.03 to 0.50, *p* = 0.03, *I*^2^ = 82.0%).

#### 3.4.1 Subgroup analysis comparing first session to last session of training

Subgroup analyses for study outcomes with significant results are presented in Table 1. A full description of the subgroup measures with sensitivity analyses can be found in the Supplementary material. Subgroup analyses were performed if there was at least more than one case per level in each category. All of the results shown in Table 1 reflect analyses with the influential case removed.

**Table 1:** Subgroup meta-analyses in studies reporting change from first to last training sessions.

**Significant subgroup differences with influential case removed—first vs. last session data**						
	***k***	**SMD**	**95% CI**	***P*** **-value effect in subgroup**	***P*** **-value subgroup differences**	*I* ^2^
**Device**						
EEG	25	0.40	[0.25, 0.54]	< 0.01 < 0.01	0.0095	77.30%
fMRI	9	0.19	[0.10, 0.28]			0.00%
**Rehearsal**						
No	24	0.39	[0.25, 0.54]	< 0.01	0.045	75.20%
Yes	10	0.20	[0.05, 0.35]	0.02		44.50%
**Feedback type**						
Simple Visual	15	0.16	[0.07, 0.25]	< 0.01	< 0.01	15%
Simple Audiovisual	4	0.59	[−0.01, 1.19]	0.05		62%
Simple Audio	2	0.28	[−3.19, 3.75]	< 0.49		88%
Complex	14	0.50	[0.29, 0.71]	< 0.01		75%
**EEG variables**						
**EEG target**						
Alpha	9	0.18	[−0.04, 0.41]	0.10	< 0.01	75.20%
SMR	8	0.66	[0.33, 0.98]	< 0.01		68.00%

K, number of studies; SMD, standardized mean difference (Hedges' g); *I*^2^, heterogeneity.

EEG neurofeedback led to a significantly larger training effect compared to fMRI neurofeedback (${\chi^{2}}_{1}=$ 6.73, *p* < 0.01). The lack of pre-training rehearsal led to a significantly larger training effect compared to the use of pre-training rehearsal (${{\;\chi }^{2}}_{1}=$ 4.02, *p* < 0.05). Alpha EEG neurofeedback training led to a significantly larger training effect than sensorimotor rhythm neurofeedback (SMR) (${\chi^{2}}_{1}$ = 7.83, *p* < 0.01).

There was a significant difference between various feedback modalities on training effect (${\chi^{2}}_{3}$ = 14.60*, p* < 0.01). Interventions using complex (SMD = 0.50) or audiovisual stimuli showed larger effects compared to those using visual or auditory feedback alone. Complex stimuli (SMD = 0.50, 95% CI: 0.29 to 0.71) and audiovisual stimuli (SMD = 0.59, 95% CI: −0.01 to 1.19) showed moderate-to-large effect sizes, while visual stimuli (SMD = 0.16, 95% CI: 0.07 to 0.25) and audio-only feedback (SMD = 0.28, 95% CI: −3.19 to 3.75) yielded smaller effects.

There were no significant differences in training effects between up- or downregulation of the training target (${\chi^{2}}_{1}$ = 0.70*, p* = 0.40), presence of explicitly given instructions as to how to modulate the training stimulus (${\chi^{2}}_{1}$ = 1.35*, p* = 0.24), absence or presence of a functional localizer (${\chi^{2}}_{1}=\;0.55,\;$*p* = 0.46), region of interest (${\chi^{2}}_{2}=2.04,\;p=\;0.36)$, use continuous or intermittent timing (${\chi^{2}}_{1}=1.89,\;p=0.17)$, use of blinding (${\chi^{2}}_{1}=0.03,\;p=\;0.86)$, or presence of extrinsic motivation (${\chi^{2}}_{1}=0.35,\;p=0.55)$.

#### 3.4.2 Subgroup analysis comparing pre-training baseline to post-training rest

When comparing pre-training baseline to post-training rest, it was found that upregulation of the training target signal led to a significantly larger training effect compared to downregulation (${\chi^{2}}_{1}$ = 5.59, *p* = 0.02).

There was no significant difference in training effect between EEG band targets (${\chi^{2}}_{2}$ = 1.21*, p* = 0.55), presence or absence of explicit instruction (${\chi^{2}}_{1}$= 0.05, *p* = 0.82), categories of stimulus presented (${\chi^{2}}_{3}$= 1.75*, p* = 0.63), presence of extrinsic motivation (${\chi^{2}}_{1}=0.68,\;p=\;0.41)$, use of blinding (${\chi^{2}}_{1}=0.08,\;p=\;0.77)$, or regions of interest (${\chi^{2}}_{2}$= 1.83*, p* = 0.40).

#### 3.4.3 Meta-regression

In studies that reported the change between first and last training trials, total training duration (log transformed) significantly moderated standardized mean difference, but only when the influential case was removed (β = 0.085, *95% CI* 0.02 to 0.15, *R*^2^ = 0.13, *p* = 0.02). Total training duration (log transformed) did not moderate SMD from baseline to post-training rest. Age did not moderate SMD. A summary of all meta-regression analyses is presented in Supplementary material

#### 3.4.4 Publication bias

Studies reporting change from first training trial and change from baseline were assessed separately for publication bias. According to visual inspection of the funnel plot (Supplementary material) and Egger's regression test there is significant publication bias in studies reporting change from the first neurofeedback training trial (β = 2.45, *95% CI*: 0.2 to 4.7, *t* = 2.13, *p* = 0.04). In studies reporting change from baseline, visual inspection of the funnel plot and Egger's test results indicated that there is no significant evidence of bias (β = 2.77, *95% CI*: −0.22 to 5.77, *t* = 1.82, *p* = 0.09).

## 4 Discussion

This meta-analysis examined the relationship between neurofeedback training design parameters and neural outcomes. While previous work has compared various neurofeedback parameters in isolation or qualitatively, this meta-analysis is the first to quantify this relationship in a non-clinical population. The primary aim of the study was to evaluate how various neurofeedback parameters influence neural modulation. To make use of all available reported data, modulation was measured in two separate ways: from first to last training session, and from pre-training baseline to post-training rest. Results showed that neurofeedback training led to significant changes in neural modulation for both measures of neural modulation. The neurofeedback imaging device used, complexity of the feedback stimulus, presence of a pre-training rehearsal trial, and EEG target oscillations, significantly contributed to modulation from first training session to the last training session. When comparing pre-training baseline to post-training rest, directionality of regulation (up or downregulation) was found to be a significant contributor to modulation of neural activity.

### 4.1 Total training duration moderates signal change after neurofeedback

Total training time influenced how much neural activity changed between the first and last training sessions—studies with longer training periods showed greater changes, following a linear relationship after log-transformation. However, no clear inflection point or plateau, so the current study was not able to specify an optimal training duration. When analyzing changes in resting state activity from before to after training, the current meta-analysis indicated that total neurofeedback training duration did not affect changes in neural modulation.

### 4.2 Device type and EEG target oscillations significantly influence observed effect sizes

After removing one outlier study, EEG studies showed a significantly higher overall effect size compared to fMRI studies. EEG studies also showed much greater heterogeneity in their results (*I*^2^ = 77%) than fMRI studies, which had no heterogeneity (*I*^2^ = 0%), indicating that variability observed in fMRI results was not due to between-study variability but due to sampling error. To better understand factors contributing to this heterogeneity, the current study examined several EEG-specific factors: the frequency band targeted, the number of feedback channels used, and whether one or multiple frequency bands were trained. Of these, only the target frequency band significantly contributed to the differences between studies. Most EEG studies (85%) focused on sensory-motor response (SMR), alpha, or beta bands. Other frequency bands were targeted in one study and were therefore not included in the statistical analysis.

The current results indicate that training aimed at SMR (12–15 Hz) produced greater signal changes across training sessions than training aimed at alpha (8–12 Hz). This finding is consistent with Gong et al. (2020), which reported that while participants could learn to modulate both SMR and alpha, SMR training produced larger power increases compared to rest. In contrast, alpha training led to more pronounced changes in resting-state alpha power. These results suggest that alpha neurofeedback training may have a stronger impact on resting-state brain activity, while SMR and beta neurofeedback training effects are more noticeable during active training. This in total is further evidence that success of neurofeedback may be context-dependent. It is also important to note, however, that some studies report more generalized effects of neurofeedback training. Bagherzadeh et al. (2020) for example, has found that training alpha synchrony in the parietal cortex led to changes in gamma synchrony in the parietal cortex and changes in alpha synchrony in the visual cortex.

### 4.3 Effects of pre-training rehearsal on signal change

In contrast to our hypothesis, the current results show that incorporating a pre-training rehearsal led to a smaller effect size compared to those without rehearsal. Pre-training rehearsals are practice sessions where participants attempt to modulate their brain signals, intended to help them acclimate to the task and lab environment. However, in this meta-analysis which focused on changes from first to last session, rehearsals may have elevated activity during the first session, thereby reducing the observed change between the first and last sessions of neurofeedback training.

In contrast to the current study, Haugg et al. (2021) identified pre-training rehearsals as a significant predictor of neurofeedback success—defined as modulating neural signals in the intended direction on more than 50% of trials. Unlike the current study, which focused on EEG and fMRI in non-clinical adults, Haugg et al. focused solely on fMRI studies in clinical patients. It is possible that pre-training rehearsals facilitate fMRI training but impair EEG training due to fundamental differences between the two modalities. fMRI neurofeedback is inherently delayed by the hemodynamic response, so participants may require more time to learn the mapping between their mental strategy and the feedback signal; in that context, a rehearsal does not diminish most of the training benefit. In contrast, EEG feedback is immediate and therefore pre-training rehearsal may inflate participants' initial performance.

### 4.4 Feedback type

Neurofeedback displays in the present meta-analysis were categorized into four types: simple audio, simple visual, simple audiovisual, and complex. Simple audio feedback refers to auditory-only cues—typically tones or beeps—that vary in pitch or volume to reflect ongoing neural activity. Simple visual feedback relies exclusively on minimally animated visuals, such as bars or line graphs, to convey real-time changes in the targeted brain signal. Simple audiovisual feedback combines these approaches by pairing a basic visual representation (for instance, a bar graph) with a synchronized auditory cue, thereby engaging two sensory channels simultaneously. Finally, complex feedback involves dynamic, interactive stimuli—often including animations, gamified interfaces, or richly layered audiovisual scenes—that present neural information through multiple, integrated feedback elements.

When comparing these modalities, complex feedback yielded the largest within-training improvements in neural modulation, indicating that richer, multi-element displays facilitate more robust learning. In contrast, simple visual feedback produced the smallest effect sizes. Taken together, these findings demonstrate the importance of feedback richness: complex, multi-element displays appear most effective for facilitating neurofeedback training.

### 4.5 Other parameters

The current meta-analysis did not identify additional parameters that significantly explained variability in neurofeedback outcomes. However, this absence of significant effects may reflect a limitation in data availability rather than a true lack of difference. Several subgroup comparisons were based on a small number of studies, making it difficult to detect meaningful differences even if they exist.

One area where this limitation was especially clear is the timing of feedback delivery, in which case only two out of forty-five studies used intermittent feedback, compared to forty-three studies which used continuous feedback. In operant conditioning, reinforcement schedules can be continuous—where feedback is given immediately after each behavior—or intermittent, where feedback is delivered intermittently with a fixed or varied schedule (Skinner and Morse, 1958). In neurofeedback, continuous feedback is commonly assumed to be more effective, under the premise that real-time reflection of brain activity allows participants to adjust their behavior more precisely. However, this assumption has been challenged. For example, Hellrung et al. (2018) found that intermittent feedback produced greater signal changes than continuous feedback, arguing that real-time feedback might demand excessive cognitive resources, forcing participants to divide attention between monitoring feedback and regulating their brain activity. Intermittent feedback, by contrast, may reduce cognitive load and foster a more focused internal regulation process. The current meta-analysis was not able to include enough studies using intermittent feedback to make a meaningful comparison with studies using continuous feedback.

Feedback timing may also contribute to observed differences between EEG and fMRI-based neurofeedback. While EEG supports real-time feedback, fMRI involves intrinsic delays due to the slower hemodynamic response and image acquisition process. If intermittent feedback proves advantageous in certain contexts, these delays in fMRI might align more closely with effective reinforcement timing. The current meta-analysis detected a much lower variability in neurofeedback training effects in fMRI studies compared to EEG studies, which might partly be based on differences in feedback timing.

### 4.6 Limitations

There are several limitations of the current meta-analysis. First, the meta-analysis focused exclusively on non-clinical populations, limiting the generalizability of these findings to clinical populations. Clinical populations often demonstrate greater room for improvement and, consequently, more pronounced neural changes as a result of neurofeedback training. For example, Haugg et al. (2021) reported that clinical populations showed significantly higher success ratios in neurofeedback interventions compared to non-clinical groups, highlighting the need for studies that include diverse participant samples.

Second, there was inconsistency in how neural change was measured across studies. While some studies reported within-training outcomes—such as differences between the first and last training sessions—others reported only pre- to post-intervention changes. These outcome types are not equivalent and may capture different aspects of the learning and consolidation process. Of the 55 included groups, 35 provided within-training data, while 20 provided only pre-post comparisons. This heterogeneity complicates the interpretation and comparison of effects across studies. Additionally, overall, very few studies reported quantifiable data useful for meta-analysis, resulting in many studies being excluded during the filtering process due to insufficient data.

Third, not all parameter categories could be fully analyzed due to incomplete or uneven reporting across the literature. Certain key variables were missing from a substantial number of studies, reducing the statistical power and limiting subgroup analyses. Even among analyzable parameters, the number of groups available for comparison was often small, reducing confidence in the generalizability of parameter-specific findings.

Fourth, evidence for publication bias was found in studies that reported change from first to last training session, indicating that the interpretation of the results should be approached with caution. The field should take account of the potential publication bias present Journals can combat this by publishing high quality research regardless of its outcome. More replication studies are also needed to counteract the possible overestimation of effect sizes.

The use of Hedges' *g* helped correct for bias in smaller sample sizes but it does not address all potential statistical concerns, such as violations of the assumption of normality or heterogeneity of variance across studies. Finally, some parameter implementations were so infrequently used that they had to be excluded altogether. While this approach ensured analytical reliability, it may have inadvertently excluded promising experimental techniques that are still emerging within the field. We recommend future studies to follow the CRED-NF reporting checklist to increase consistency across different studies (Ros et al., 2020).

Additionally, this meta-analysis did not account for individual baseline neurophysiological factors such as menstrual cycle phase (Bazanova et al., 2017), dominant EEG frequency patterns, psycho-emotional tension (e.g., EMG) (Goncharova et al., 2003), or ergonomic conditions like eye state (i.e., open or close) and postural support. These variables could influence both the effectiveness of neurofeedback and the quality of the recorded signals. To improve comparability and interpretability across studies, future research should systematically record and report such factors.

Finally, the current study was not able to perform analyses related to retention since there were insufficient studies in our sample reporting follow-up sessions after initial training. In order to understand the long-lasting effects of neurofeedback future studies should incorporate follow-up sessions in their study designs.

## 5 Conclusion

The purpose of this meta-analysis was to elucidate factors that contribute to successful neurofeedback training. Overall, it was found that neurofeedback exerts a small but significant effect on neural activity, with effect sizes being larger when neural modulation was measured from first to last training sessions compared to resting-state assessments made before and after training. This distinction suggests that neurofeedback may be more effective in altering neural dynamics during active engagement than in producing enduring changes observable at rest.

Among the parameters examined, total training duration, EEG target frequency, feedback type, device modality, and the presence of pre-training rehearsal emerged as meaningful factors in neural modulation, although their impact varied by outcome type. For example, longer training durations improved modulation during sessions but had no significant impact on resting-state changes. Similarly, the SMR frequency band appeared to support stronger signal changes during training, while alpha training was more associated with resting-state effects—emphasizing the importance of aligning the neurofeedback protocol with the desired neural or cognitive outcome.

The current findings also underscore the influence of feedback design: complex and multimodal feedback were associated with larger effect sizes, suggesting that participant engagement and the richness of feedback cues may enhance the learning process.

The findings from this meta-analysis point to the need for a more rigorous and standardized approach to reporting neurofeedback research. Greater adherence to frameworks such as the CRED-NF checklist (Ros et al., 2020) would facilitate data synthesis, reduce bias, and support the identification of best practices. Moreover, the high heterogeneity observed in EEG studies—compared to fMRI—suggests that methodological refinement and consistency are especially critical in EEG-based research.

## Data Availability

The original contributions presented in the study are included in the article/Supplementary material, further inquiries can be directed to the corresponding author.
