# Supplementary material for: Systematic review and meta-analysis of the relationships between real-time neurofeedback training parameters and acquisition of neural modulation
Source: Front Hum Neurosci. 2025 Aug 29;19:1652607. doi: 10.3389/fnhum.2025.1652607 (PMC12426165; doi:10.3389/fnhum.2025.1652607)
Supplement: Supplementary file 1 [file Data_Sheet_1.docx]

## Data Abstraction Form

**Relationships between Neurofeedback Training Parameters and Acquisition and Retention of Neural Modulation: A Protocol for Meta-Analysis**

Study ID:

Date of Abstraction: ____/____/____

Initials of Data Abstractor: ________

General Information

1. Article title: _____________________________________________
2. First author (last name, first initial): __________________________
3. Publication year: _________________________________________
4. Journal title: ____________________________________________
5. Study country: ___________________________________________

6. Blinding

1. Open
2. Single (participant)
3. Double (participant, investigator)
4. Triple (participant, investigator, analyst)

7. Neuroimaging device

1. fNIRS
2. fMRI
3. EEG
4. MEG

8. Neurotypical/control neurofeedback training group sample size after exclusions

1. ___________ participants

9. Sex of neurofeedback training group before exclusions

1. ___________ males

2. ___________ females

10. Sex of neurofeedback training group after exclusions

1. ___________ males

2. ___________ females

11. Average age of neurofeedback training group before exclusions

1. Mean: ___________

2. Standard deviation: _____________

12. Average age of neurofeedback training group after exclusions

1. Mean: ___________

2. Standard deviation: _____________

13. Strategy used by participants to develop control of neurofeedback stimulus

1. Auditory (specify)
2. Visual (specify)
3. Cognitive (specify)
4. Emotional (specify)
5. Verbal (specify)
6. Motor (specify)
7. None (specify)
8. Other (specify)

14. Presence of explicit instructions given to develop control

- 1. Yes, describe with examples: _______
- 2. None

15. Presence of extrinsic motivation

- 1. Yes, describe: _______

1. None

16. Feedback display

1. Auditory feedback
2. Cue size
3. Facial expression
4. Line/bar graph
5. Meter/thermometer
6. Overlaid image opacity
7. Reward image
8. Number/feedback value
9. Resolution of stimulus
10. Color differences
11. Brightness differences
12. Dynamic goal-oriented stimulus
13. Other (specify)

17. Neurofeedback stimulus presentation data collection bin

1. Describe: _______________

18. Neurofeedback stimulus presentation update timing

1. Continuous (specify any delay in updating stimulus, if provided)
2. Intermittent (specify duration)

19. Task demands between neurofeedback training intervals

1. Type
   1. Active task (describe)
   2. Passive task (describe)
   3. Both (describe)
   4. None
2. Frequency
   1. Describe: ____________
3. Duration
   1. _________ seconds

*Repeat parameter 19 for each additional task as needed

17. Duration of neurofeedback trials during acquisition

1. ____________ seconds

18. Inter-trial interval between neurofeedback trials during acquisition

1. ____________ seconds

19. Neurofeedback trials per block during acquisition

1. ____________ trials

20. Inter-block interval during acquisition

1. _____________ seconds

21. Blocks per session during acquisition

1. ____________ blocks

22. Inter-session interval during acquisition

1. ____________ days

23. Number of sessions during acquisition

1. _____________ sessions

24. Duration of session during acquisition

1. _____________ minutes

25. Total training duration during acquisition

1. _____________ days

26. Total time spent neurofeedback training

1. _____________ minutes

27. Presence of a transfer run?

1. Yes, describe duration: _______ seconds

2. No

28. Activation change baseline determination during acquisition

1. Baseline from initial trial
2. Baseline continuously updated after activation
   1. Between trials
   2. Between blocks
   3. Between sessions
   4. Other __________
3. Not reported
4. Other (describe)

29. BOLD signal modulation of a specific ROI

1. Upregulation
2. Downregulation
3. Upregulation and downregulation
   1. Trials/blocks/sessions before change (describe)
   2. Number of changes (describe)
4. Lateralization/network asynchrony
5. Lateralization/network functional connectivity
6. Other (specify)

30. EEG modulation

A. Modulation of amplitude of a specific signal

1. Upregulation

2. Downregulation

3. Upregulation and downregulation

A. Trials/blocks/sessions before change (describe)

1. Number of changes (describe)

4. Not applicable

B. Modulation of frequency of a specific signal

1. Tuning to neural oscillation frequency/ratio

2. Not applicable

C. Coherence

1. Increase

2. Decrease

D. Other (specify)

31. EEG neural oscillation frequency/ratio

1. Alpha (specify frequency)
2. Beta (specify frequency)
3. Gamma (specify frequency)
4. Delta (specify frequency)
5. Theta (specify frequency)
6. Multiple during time period (specify)
7. Ratio (specify)
8. Other (specify)
9. Not applicable

32. Regions targeted during feedback

A. Localized regions

- - - 1. ______________
      2. ______________
      3. ______________
      4. ______________
      5. ______________

B. Feedback electrode/channel sites

1. ______________
2. ______________
3. ______________
4. ______________
5. ______________

C. Network nodes

1. ______________
2. ______________
3. ______________
4. ______________
5. ______________

D. Networks implicated in coherence/asynchronous measures

If D. Specify network name: ______________

1. Network 1 ROIs: _________

2. Network 2 ROIs: _________

3. Network 3 ROIs: _________

4. Others: _________________

33. Regions of measured signal change

A. Localized regions

1. ______________
2. ______________
3. ______________
4. ______________
5. ______________

B. Feedback electrode/channel sites

1. ______________
2. ______________
3. ______________
4. ______________
5. ______________

C. Network nodes

1. ______________
2. ______________
3. ______________
4. ______________
5. ______________

D. Networks implicated in coherence/asynchronous measures

1. Network 1 ROIs: _________

2. Network 2 ROIs: _________

3. Network 3 ROIs: _________

4. Others: _________________

34. Regional specificity

1. Voxel coordinates ___________

a. Coordinate system (MNI, Talairach) _________

2. Size of voxel ____________ cubic mm

3. Volume of the ROI used in neurofeedback training (cm^3^)

35. Use of pre-training rehearsal prior to neurofeedback intervention?

1. Yes, describe frequency: _______, describe duration: ___________

2. No

36. Use of functional localizers to determine regions of interest?

1. Yes, Describe: ________
2. No

37. Acquisition test signal change from baseline

1. Signal change value: _________ specify change
   1. Percent signal change: _________
   2. Percent change standard deviation: ________
   3. Beta-values: _________
2. Other information to calculate variance
   1. Standard error: ___________
   2. T-values: ____________
   3. 95% confidence intervals: ___________
   4. Other: ___________

*Repeat parameter #37 for each individual run if applicable

38. Interval between acquisition and retention

1. _________ days

39. Duration of neurofeedback trials during retention

1. _________ seconds

40. Intertrial interval during retention

1. _________ seconds

41. Trials per block during retention

1. __________ trials

42. Interblock interval during retention

1. _____________ seconds

43. Blocks per session during retention

1. ____________ blocks

44. Intersession interval during retention

1. ____________ days

45. Number of sessions during retention

1. _____________ sessions

46. Duration of session during retention

1. _____________ minutes

47. Total training duration during retention

1. _____________ days

48. Retention test signal change from baseline

1. Signal change value: _________ specify change
   1. Percent signal change: _________
   2. Percent change standard deviation: ________
   3. Beta-values: _________
2. Other information to calculate variance
   1. Standard error: ___________
   2. T-values: ____________
   3. 95% confidence intervals: ___________
   4. Other: ___________

*Repeat parameter #48 for each individual run if applicable

49. Activation change baseline determination during retention

1. Baseline from first retention trial
2. Baseline continuously updated after activation
   1. Between trials
   2. Between blocks
   3. Between sessions
   4. Other __________
3. Not reported
4. Other (describe)

50. Transfer run signal change from baseline

1. Signal change value: _________ specify change
   1. Percent signal change: _________
   2. Percent change standard deviation: ________
   3. Beta-values: _________
2. Other information to calculate variance
   1. Standard error: ___________
   2. T-values: ____________
   3. 95% confidence intervals: ___________
   4. Other: ___________

49. Transfer run activation change baseline determination

1. Baseline from first retention trial
2. Baseline continuously updated after activation
   1. Between trials
   2. Between blocks
   3. Between sessions
   4. Other __________
3. Not reported
4. Other (describe)

51. Definition of neurofeedback success: acquisition

1. Define: ________

52. Definition of neurofeedback success: retention

1. Define: ________

53. Neurofeedback success rate: acquisition

1. _________ %

54. Neurofeedback success rate: retention

1. _________ %
