# Supplementary material for: Systematic review and meta-analysis of the relationships between real-time neurofeedback training parameters and acquisition of neural modulation
Source: Front Hum Neurosci. 2025 Aug 29;19:1652607. doi: 10.3389/fnhum.2025.1652607 (PMC12426165; doi:10.3389/fnhum.2025.1652607)

# Figure S2. Egger’s Test

Figure S2a. Funnel plot showing the relationship between standard error and standardized mean difference (Hedges’ g) in studies showing difference between first and last training trials.


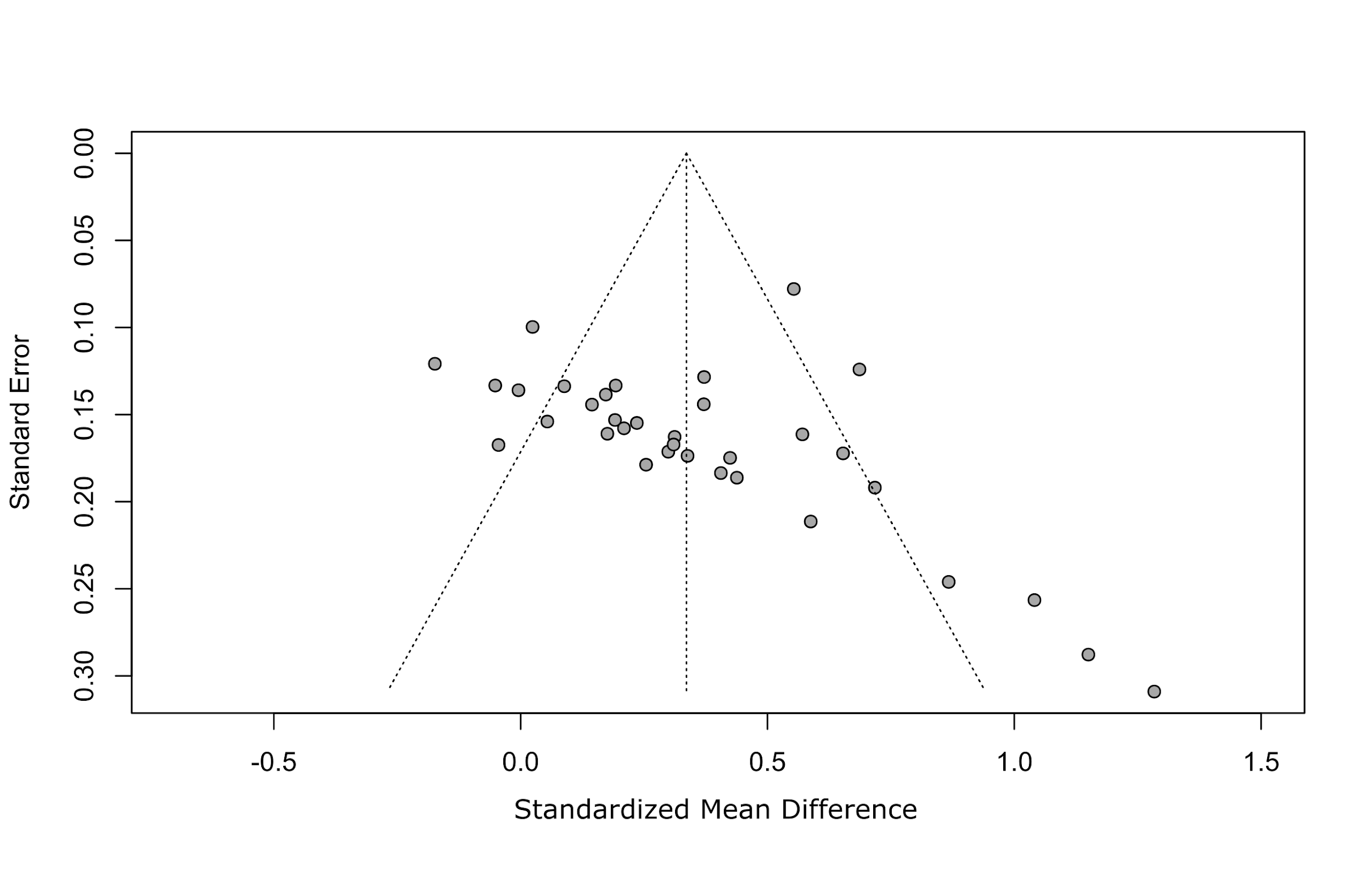


Figure S2b. Funnel plot showing the relationship between standard error and standardized mean difference (Hedges’ g) in studies showing difference between pre-training baseline and post-training rest.


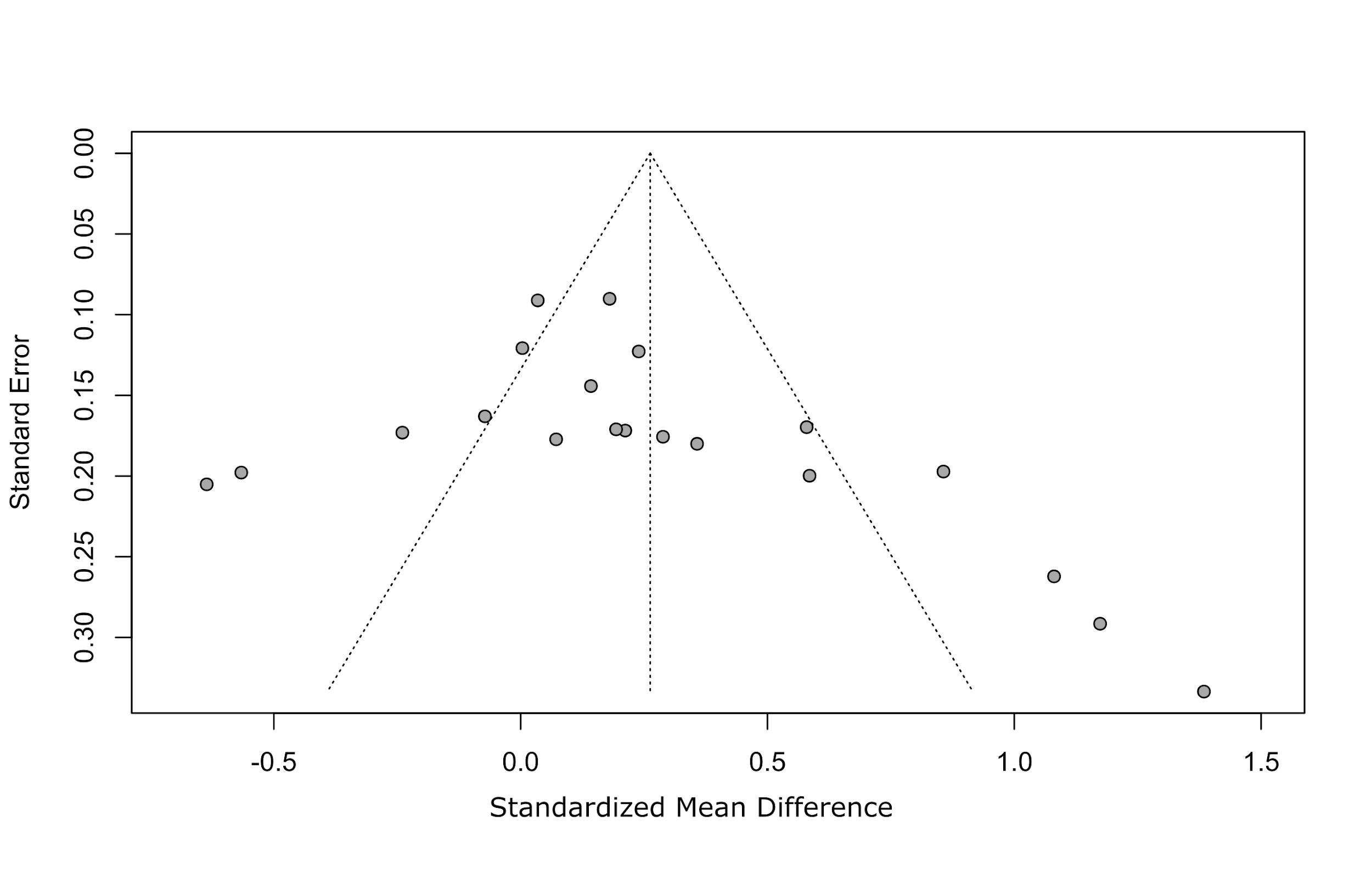

Supplement: Supplementary file 2 [file Data_Sheet_2.docx]
