# Supplementary material for: Systematic review and meta-analysis of the relationships between real-time neurofeedback training parameters and acquisition of neural modulation
Source: Front Hum Neurosci. 2025 Aug 29;19:1652607. doi: 10.3389/fnhum.2025.1652607 (PMC12426165; doi:10.3389/fnhum.2025.1652607)
Supplement: Supplementary file 3 [file Table_1.docx]

Supplementary Material

# Supplementary Data. Study Characteristics

Table S1. Extracted data from the studies included in the meta-analysis

| *Author* | *Group* | *Citation* | *n* | *Mean Age* | *Device* | *Blinding* | *Instruction provided* | *Feedback Update Timing* | *Pre-training Rehearsal* | *Functional Localizer* |
| --- | --- | --- | --- | --- | --- | --- | --- | --- | --- | --- |
| Alexander |  | Alexander et al., 2018 | 16 | 46.66±8.1 | EEG | No | No | CONT | No | Yes |
| Anil |  | Anil et al., 2022 | 25 | 30.96±11.19 | EEG | No | No | CONT | No | No |
| Cannon |  | Cannon et al., 2014 | 5 | N/A | EEG | No | No | CONT | No | No |
| Cheng |  | Cheng et al., 2015 | 8 | 20.6±1.59 | EEG | Yes | No | CONT | No | No |
| Christie |  | Christie et al., 2020 | 8 | N/A | EEG | No | No | CONT | No | No |
| Dekker |  | Dekker et al., 2014 | 18 | 20.7±1.79 | EEG | No | No | CONT | No | No |
| Dempster |  | Dempster and Vernon, 2009 | 25 | N/A | EEG | No | No | CONT | No | No |
| deZambotti |  | deZambotti et al., 2012 | 8 | 23.12±1.8 | EEG | No | Yes | CONT | No | No |
| Domingos_1 | 2 session/ week | Domingos et al., 2021 | 13 | N/A | EEG | No | Yes | CONT | Yes | Yes |
| Domingos_2 | 3 session/week | Domingos et al., 2021 | 14 | N/A | EEG | No | Yes | CONT | Yes | Yes |
| Emmert_1 | AIC | Emmert et al., 2014 | 14 | 27.6±2.1 | fMRI | No | No | CONT | No | Yes |
| Emmert_2 | ACC | Emmert et al., 2014 | 14 | 27.4±2.6 | fMRI | No | No | CONT | No | Yes |
| Gadea |  | Gadea et al., 2020 | 16 | N/A | EEG | Yes | No | CONT | No | No |
| Gadea_2 |  | Gadea et al., 2016 | 10 | N/A | EEG | Yes | No | CONT | No | No |
| Gevensleben |  | Gevensleben et al., 2014 | 9 | 23.2±2.91 | EEG | Yes | Yes | CONT | No | No |
| Goksin |  | Goksin et al., 2019 | 11 | 29±3.04 | EEG | No | Yes | CONT | No | No |
| Guleken |  | Guleken et al., 2020 | 8 | N/A | EEG | No | No | CONT | No | No |
| Hellrung_1 | Continuous | Hellrung et al., 2018 | 16 | 25.8±2.4 | fMRI | Yes | Yes | CONT | Yes | Yes |
| Hellrung_2 | Intermittent | Hellrung et al., 2018 | 18 | 27.8±3.8 | fMRI | Yes | Yes | INT | Yes | Yes |
| Hoedlmoser |  | Hoedlmoser et al., 2008 | 16 | N/A | EEG | Yes | No | CONT | No | No |
| Keynan_1 | Amyg-EFP | Keynan et al., 2019 | 38 | 21±NA | EEG | Yes | No | CONT | No | No |
| Keynan_2 | EEG | Keynan et al., 2019 | 88 | 21±NA | EEG | Yes | No | CONT | No | No |
| Kober_1 | Upregulation | Kober et al., 2020 | 10 | 24.5±2.22 | EEG | No | Yes | CONT | No | No |
| Kober_2 | Downregulation | Kober et al., 2020 | 10 | 25.3±2.16 | EEG | No | Yes | CONT | No | No |
| Kober_3 |  | Kober et al., 2022 | 13 | 29.31±5.27 | EEG | Yes | No | CONT | Yes | No |
| Kober_4 |  | Kober et al., 2022 | 13 | 27.62±5.52 | EEG | Yes | No | CONT | Yes | No |
| Kober_5 |  | Kober et al., 2013 | 10 | 46±3.98 | EEG | No | No | CONT | Yes | No |
| Krogmeier |  | Krogmeier et al., 2022 | 19 | 19.05±1.02 | EEG | No | Yes | CONT | Yes | No |
| Maszczyk |  | Maszczyk et al., 2018 | 8 | N/A | EEG | Yes | No | CONT | No | No |
| Mayeli |  | Mayeli et al., 2020 | 18 | N/A | fMRI | No | Yes | CONT | Yes | No |
| Miroslaw | Swimmers | Mirosław and Grzegorz, 2022 | 10 | 21.5±NA | EEG | No | No | CONT | No | No |
| Miroslaw_2 | Track and Field | Mirosław and Grzegorz, 2022 | 10 | 21.5±NA | EEG | No | No | CONT | No | No |
| Naas |  | Naas et al., 2019 | 17 | 21.29±NA | EEG | Yes | Yes | CONT | No | Yes |
| Navarro |  | Navarro et al., 2018 | 24 | 37.1±11.1 | EEG | No | No | CONT | No | No |
| Patel |  | Patel et al., 2021 | 4 | N/A | EEG | No | No | CONT | No | No |
| Perez-Elvira_1 |  | Perez-Elvira et al., 2021 | 48 | 35.9±12.02 | EEG | Yes | No | CONT | No | No |
| Perez-Elvira_2 |  | Perez-Elvira et al., 2021 | 45 | 38.42±12.17 | EEG | Yes | No | CONT | No | No |
| Pimenta_1 | SMR | Pimenta et al., 2018 | 10 | 23±1.45 | EEG | Yes | No | CONT | No | No |
| Pimenta_2 | Beta | Pimenta et al., 2018 | 11 | 21.91±1.35 | EEG | Yes | No | CONT | No | No |
| Rijken |  | Rijken et al., 2016 | 10 | 18±NA | EEG | No | No | CONT | No | No |
| Shibata_1 | Low Preference | Shibata et al., 2016 | 12 | N/A | fMRI | Yes | No | INT | No | Yes |
| Shibata_2 | High Preference | Shibata et al., 2016 | 12 | N/A | fMRI | Yes | No | INT | No | Yes |
| Staufenbiel_1 | Gamma | Staufenbiel et al., 2014 | 10 | 66.4±1.9 | EEG | Yes | No | CONT | No | No |
| Staufenbiel_2 | Beta | Staufenbiel et al., 2014 | 10 | 69.2±1.87 | EEG | Yes | No | CONT | No | No |
| Studer_1 | SCP | Studer et al., 2014 | 19 | 25.08±2.47 | EEG | No | No | CONT | No | No |
| Studer_2 | Theta/Beta | Studer et al., 2014 | 19 | 24.62±2.56 | EEG | No | No | CONT | No | No |
| Tribrat |  | Tribrat et al., 2007 | 37 | N/A | EEG | Yes | Yes | CONT | No | No |
| vanSon_1 | 14 Sessions | vanSon et al., 2020 | 4 | 20.5±1 | EEG | No | No | CONT | No | No |
| vanSon_2 | 8 Sessions | vanSon et al., 2020 | 4 | 21.25±1.25 | EEG | No | No | CONT | No | No |
| Weiss |  | Weiss et al., 2022 | 20 | N/A | fMRI | Yes | No | CONT | No | No |
| Mehran |  | Mehran et al., 2013 | 8 | N/A | EEG | Yes | Yes | CONT | No | No |
| Zhang |  | Zhang et al., 2013 | 12 | 21±2 | fMRI | No | Yes | CONT | Yes | Yes |

# Supplementary Data. Study Characteristics (continued)

| *Author* | *Direction* | *Feedback Type* | *Training Duration (days)* | *Total Training Duration (min)* | *Freq. Band* | *ROI* | *Initial Value* | *g* | *95% CI* | *Weight* |
| --- | --- | --- | --- | --- | --- | --- | --- | --- | --- | --- |
| Alexander | up | audio | 1 | 15 | alpha | O1 | Baseline | 0.86 | [0.47; 1.24] | 4.9 |
| Anil | up | bar | 28 | 120 | alpha | C4 | First NF Trial | -0.17 | [-0.41; 0.06] | 3.3 |
| Cannon | up | avatar/game | 16-28 | 480 | alpha | Precuneus | Baseline | 1.38 | [0.73; 2.04] | 3.7 |
| Cheng | up | audio + bar | 35 | 48 | SMR | Cz | First NF Trial | 0.59 | [0.17; 1.00] | 2.5 |
| Christie | up | Audio + visual | 137 | 1350 | SMR | Cz | First NF Trial | 1.28 | [0.68; 1.89] | 1.8 |
| Dekker | up | audio | 19 - 28 | 360 | alpha | C3, C4 | First NF Trial | 0.57 | [0.25; 0.89] | 2.9 |
| Dempster | up | bar and/or audio | 70 | 150 | alpha | Pz | Baseline | 0.37 | [0.12; 0.62] | 3.2 |
|  |  |  |  |  |  |  | First NF Trial | 0.24 | [-0.00; 0.48] | 5.5 |
| Domingos_1 | up | object changing shape and position | 42 | 300 | alpha | Cz | First NF Trial | 0.05 | [-0.25; 0.36] | 3 |
| Domingos_2 | up | object changing shape and position | 28 | 300 | SMR | Cz | First NF Trial | 0.72 | [0.34; 1.09] | 2.7 |
| deZambotti | up | game | 56 | 240 | SMR/theta | FCz | First NF Trial | 0.87 | [0.38; 1.35] | 2.2 |
| Emmert_1 | down | line | 1 | 8 | N/A | AIC | First NF Trial | 0.24 | [-0.07; 0.54] | 3 |
| Emmert_2 | down | line | 1 | 8 | N/A | ACC | First NF Trial | 0.19 | [-0.11; 0.49] | 3 |
| Gadea | up | video (activate) | 1 | 30 | SMR | Cz | Baseline | 0.58 | [0.25; 0.91] | 5.1 |
| Gadea_2 | up | video (activate) | 1 | 30 | SMR | C3 | Baseline | 0.21 | [-0.12; 0.55] | 5.1 |
| Gevensleben | up | bar | 21 | 192 | SCP | Cz | First NF Trial | 0.25 | [-0.10; 0.60] | 2.8 |
| Göksin | up | bar +audio | 10 | 56.25 | alpha | P8 | Baseline and First NF Trial | 0.34 | [-0.00; 0.68] | 2.8 |
| Guleken | up | bar | 35 | 300 | SMR | C4 | First NF Trial | 1.15 | [0.59; 1.71] | 1.9 |
| Hellrung_1 | up | bar | 1 | 8 | N/A | L. Amygdala | First NF Trial | 0.11 | [-0.17; 0.40] | 3.1 |
| Hellrung_2 | up | bar | 1 | 8 | N/A | L. Amygdala | First NF Trial | 0.14 | [-0.13; 0.41] | 3.1 |
| Hoedlmoser | up | needle | 10 | 240 | SMR | C3 | Baseline | 0.14 | [-0.14; 0.43] | 5.3 |
| Keynan_1 | down | game | 28 | 30 | N/A | Pz | First NF Trial | 0.64 | [0.40; 0.88] | 3.3 |
| Keynan_2 | down | game | 28 | 30 | N/A | O1, O2, Oz | First NF Trial | 0.52 | [0.37; 0.67] | 3.6 |
| Kober_1 | up | bar | 21-28 | 180 | SMR | Cz, CPz | Baseline | 1.08 | [0.57; 1.59] | 4.3 |
| Kober_2 | down | bar | 21-28 | 180 | SMR | Cz, CPz | Baseline | -0.64 | [-1.04; -0.23] | 4.8 |
| Kober_3 | up | bar | 1 | 27 | SMR | Cz | First NF Trial | 0.21 | [-0.10; 0.52] | 2.9 |
| Kober_4 | up | bar | 1 | 27 | SMR | Cz | First NF Trial | 0.31 | [-0.01; 0.63] | 2.9 |
| Kober_5 | up | bar+audio | 21 | 180 | SMR | Cz | First NF Trial | 0.41 | [0.05; 0.77] | 2.7 |
| Krogmeier | up | game | 1 | 4 | alpha | F3, F4 | First NF Trial | -0.05 | [-0.31; 0.21] | 3.2 |
| Maszczyk | up | game | 35 | 375 | beta | O1, O2 | Baseline | 1.17 | [0.60; 1.75] | 4.1 |
| Mayeli | up | bar | 1 | 8 | beta | vmpfc | Baseline | 0.00 | [-0.27; 0.26] | 3.1 |
| Mehran | up | game | 20 | 100 | Beta | Cz | Baseline and First NF Trial | 0.07 | [-0.28; 0.42] | 5.1 |
| Miroslaw | down | game | 120 | 600 | beta | C3, C4 | Baseline | -0.57 | [-0.95; -0.18] | 4.9 |
| Miroslaw_2 | down | game | 120 | 600 | beta | C3, C4 | Baseline | 0.19 | [-0.14; 0.53] | 5.1 |
| Naas | up | color change | 4 | 60 | alpha | P7, O1, O2, P8 | First NF Trial | 0.65 | [0.32; 0.99] | 2.8 |
| Navarro | up | color change | 21 | 120 | alpha | parieto-occipital | Baseline | 0.00 | [-0.23; 0.24] | 5.5 |
| Patel | up | dial | 21 | 50 | alpha | AF3, AF4 | First NF Trial | -0.04 | [-0.37; 0.28] | 2.9 |
| Perez-Elvira_1 | up | video | 1 |  | SMR | Cz | Baseline | 0.18 | [0.00; 0.36] | 5.6 |
| Perez-Elvira_2 | up | game | 1 |  | SMR | Cz | Baseline | 0.03 | [-0.14; 0.21] | 5.6 |
| Pimenta_1 | up | game | 14-21 | 120 | SMR | C4 | First NF Trial | 1.04 | [0.54; 1.54] | 2.1 |
|  |  |  |  |  |  |  | Baseline | 0.29 | [-0.06; 0.63] | 5.1 |
| Pimenta_2 | up | game | 14-21 | 120 | beta | C3 | First NF Trial | 0.30 | [-0.04; 0.63] | 2.8 |
|  |  |  |  |  |  |  | Baseline | -0.07 | [-0.39; 0.25] | 5.2 |
| Rijken | up | audio | 42 | 444 | alpha | C3, C4 | Baseline | 0.36 | [0.00; 0.71] | 5 |
| Shibata_1 | up | bar | 3 | 117.45 | N/A | Cingulate Cortex | First NF Trial | 0.38 | [0.05; 0.72] | 2.8 |
| Shibata_2 | up | bar | 3 | 117.45 | N/A | Cingulate Cortex | First NF Trial | 0.16 | [-0.15; 0.48] | 2.9 |
| Staufenbiel_1 | up | audio | 21 | 240 | beta | Fz | Baseline | 0.59 | [0.19; 0.98] | 4.9 |
| Staufenbiel_2 | up | audio | 21 | 240 | gamma | Fz | Baseline | -0.24 | [-0.58; 0.10] | 5.1 |
| Studer_1 | up | bar | 70 | 100 | theta/beta | Cz | First NF Trial | 0.37 | [0.09; 0.65] | 3.1 |
| Studer_2 | up | bar | 70 | 1000 |  | Cz | First NF Trial | 0.09 | [-0.17; 0.35] | 3.2 |
| Tribrat | up | audio | 3 | 15 | alpha | C3, C4 | First NF Trial | 0.02 | [-0.17; 0.22] | 3.4 |
| vanSon_1 | up | video (activate) | 28-35 | 350 | theta | Frontal | First NF Trial | 0.44 | [0.07; 0.80] | 2.7 |
| vanSon_2 | up | game | 14-21 | 200 | theta | Frontal | First NF Trial | -0.85 | [-1.31; -0.40] | 2.3 |
| Weiss | up | bar | 3 | 71.125 |  | dlpfc | First NF Trial | 0.19 | [-0.07; 0.45] | 3.2 |
| Zhang | down | line | 1 | 12.27 |  | pcc | First NF Trial | 0.31 | [-0.02; 0.64] | 2.9 |

**Supplementary Data. Table S2A. Results: Change from first to last training sessions**

Subgroup meta-analyses results with and without influential case removed in studies reporting change from first to last training sessions. k = number of studies; SMD = standardized mean difference (Hedges’ g); I2= Heterogeneity.

|  | ***Full Analysis*** | | | | | ***Influential Case Removed*** | | | | |
| --- | --- | --- | --- | --- | --- | --- | --- | --- | --- | --- |
|  | ***k*** | ***SMD*** | ***95% CI*** | ***p-value (subgroup)*** | ***I^2^*** | ***k*** | ***SMD*** | ***95% CI*** | ***p-value (sub group)*** | ***I^2^*** |
| **Main Analysis** | |  |  |  |  |  |  |  |  |  |
| *First vs. Last NFT* | 35 | 0.32 | [0.19, 0.44] | <0.001* | 76.0% | 34 | 0.34 | [0.23, 0.44] | <0.001* | 71.8% |
| ***Device*** |  |  |  |  |  |  |  |  |  |  |
| *EEG* | 26 | 0.37 | [0.19, 0.54] | 0.05 | 81.10% | 25 | .40 | [0.25, 0.54] | .0095* | 77.30% |
| *fMRI* | 9 | 0.19 | [0.10, 0.28] |  | 0.00% | 9 | 0.19 | [0.10, 0.28] |  | 0.00% |
| ***Feedback Type*** |  |  |  |  |  |  |  |  |  |  |
| *Simple Visual* | 15 | 0.16 | [0.07, 0.25] | 0.04 | 14.8% | 15 | 0.16 | [0.07, 0.25] | <0.01 | 15% |
| *Simple Audiovisual* | 4 | 0.59 | [-0.01, 1.19] |  | 61.9% | 4 | 0.59 | [-0.01, 1.19] |  | 62% |
| *Simple Audio* | 2 | 0.28 | [-3.19, 3.75] |  | 88.0% | 2 | 0.28 | [-3.19, 3.75] |  | 88% |
| *Complex* | 14 | 0.42 | [0.14; 0.71] |  | 83.5% | 13 | 0.50 | [0.29, 0.71] |  | 75% |
| ***Instruction*** |  |  |  |  |  |  |  |  |  |  |
| *No* | 23 | 0.35 | [0.17, 0.52] | 0.44 | 78.50% | 22 | 0.38 | [0.24, 0.52] | 0.24 | 72.10% |
| *Yes* | 12 | 0.25 | [0.07, 0.44] |  | 66.40% | 12 | 0.25 | [0.07, 0.44] |  | 66.40% |
| ***Motivation*** |  |  |  |  |  |  |  |  |  |  |
| *No* | 4 | 0.44 | [-0.12, 1.01] | 0.45 | 64.80% | 4 | 0.44 | [-0.12, 1.01] | 0.52 | 64.80% |
| *Yes* | 31 | 0.30 | [0.17, 0.44] |  | 77.20% | 30 | 0.32 | [0.21, 0.44] |  | 73.00% |
| ***Rehearsal*** |  |  |  |  |  |  |  |  |  |  |
| *No* | 25 | 0.36 | [0.19, 0.53] | 0.13 | 79.90% | 24 | 0.39 | [0.25, 0.54] | 0.045* | 75.20% |
| *Yes* | 10 | 0.20 | [0.05, 0.35] |  | 44.50% | 10 | 0.20 | [0.05, 0.35] |  | 44.50% |
| ***Functional Localizer*** |  |  |  |  |  |  |  |  |  |  |
| *No* | 25 | 0.33 | [0.15, 0.5] | 0.70 | 81.00% | 24 | 0.36 | [0.21, 0.51] | 0.46 | 77.40% |
| *Yes* | 10 | 0.29 | [0.13, 0.44] |  | 40.30% | 10 | 0.29 | [0.13, 0.44] |  | 40.30% |
| ***Update Timing*** |  |  |  |  |  |  |  |  |  |  |
| *No* | 32 | 0.32 | [0.19, 0.46] | 0.42 | 77.80% | 31 | 0.34 | [0.23, 0.47] | 0.27 |  |
| *Yes* | 3 | 0.24 | [-0.10, 0.58] |  | 0.00% | 3 | 0.24 | [-0.10, 0.58] |  | 0.00% |
| ***Direction*** |  |  |  |  |  |  |  |  |  |  |
| *Upregulation* | 30 | 0.30 | [0.16, 0.45] | 0.33 | 74.90% | 29 | 0.32 | [0.20, 0.45] | 0.404 | 69.90% |
| *Downregulation* | 5 | 0.42 | [0.15, 0.69] |  | 62.70% | 5 | 0.42 | [0.15, 0.69] |  | 62.70% |
| ***Regions of Interest*** |  |  |  |  |  |  |  |  |  |  |
| *Frontal* | 5 | 0.07 | [-0.71, 0.85] | 0.20 | 87.3% | 4 | 0.27 | [-0.41, 0.96] | 0.36 | 79.3% |
| *Central* | 16 | 0.40 | [0.19, 0.61] |  | 76.9% | 16 | 0.39 | [0.19, 0.61] |  | 76.9% |
| *Posterior* | 5 | 0.51 | [0.37, 0.66] |  | 0.0% | 5 | 0.51 | [0.37, 0.67] |  | 0.0% |
| ***Blinding*** |  |  |  |  |  |  |  |  |  |  |
| *No* | 20 | 0.29 | [0.08, 0.49] | 0.62 | 78.9% | 19 | 0.32 | [0.15, 0.50] | 0.86 | 73.6% |
| *Yes* | 15 | 0.34 | [0.20, 0.48] |  | 66.4% | 15 | 0.34 | [0.20, 0.48] |  | 66.4% |
|  |  |  |  |  |  |  |  |  |  |  |
| ***EEG Variables*** | | | | | | | | | | |
| ***EEG Target*** |  |  |  |  |  |  |  |  |  |  |
| *Alpha* | 9 | 0.18 | [-0.04, 0.41] | <0.01 | 75.20% | 9 | 0.18 | [-0.04, 0.41] | <0.01 | 75.20% |
| *SMR* | 8 | 0.66 | [0.33, 0.98] |  | 68.00% | 8 | 0.66 | [0.33, 0.98] |  | 68.00% |

**Supplementary Data. Table S2B. Results: change from baseline to post neurofeedback training rest.**

**Table S2B.** Subgroup meta-analyses in studies reporting change from baseline to post neurofeedback training rest. k = number of studies; SMD = standardized mean difference (Hedges’ g); I2= Heterogeneity.

|  | | ***k*** | ***SMD*** | ***95% CI*** | ***p-value*** | ***I^2^*** |
| --- | --- | --- | --- | --- | --- | --- |
| **Main Analysis** |  | |  |  |  |  |
| *Baseline vs. Post-training* | | 20 | 0.26 | [0.03, 0.50] | 0.03* | 82.0% |
| ***Feedback Type*** |  |  |  |  |  |  |
| *Simple Audio* | 15 | 4 | 0.38 | [-0.36, 1.13] | 0.63 | 84.7% |
| *Simple Audiovisual* | 4 | 2 | 0.77 | [-6.49, 8.03] |  | 90.4% |
| *Complex* | 2 | 12 | 0.16 | [-0.07, 0.39] |  | 70.7% |
| *Simple Visual* | 14 | 2 | 0.21 | [-10.69; 11.12] |  | 96.2% |
| ***Instruction*** | |  |  |  |  |  |
| *No* | | 17 | 0.28 | [0.04, 0.51] | 0.82 | 78.60% |
| *Yes* | | 3 | 0.16 | [-1.96, 2.30] |  | 92.50% |
| ***Motivation*** | |  |  |  |  |  |
| *No* | | 18 | 0.28 | [0.02, 0.55] | 0.41 | 83.00% |
| *Yes* | | 2 | 0.10 | [-2.19, 2.40] |  | 55.90% |
| ***Direction*** | |  |  |  |  |  |
| *Upregulation* | | 17 | 0.35 | [0.13, 0.58] | 0.018* | 77.60% |
| *Downregulation* | | 3 | -0.33 | [-1.48, 0.83] |  | 84.30% |
| ***Regions of Interest*** | |  |  |  |  |  |
| *Frontal* | | 2 | 0.17 | [-0.32, 1.37] | 0.40 | 89.7% |
| *Central* | | 13 | 0.14 | [-0.12, 0.39] |  | 77.0% |
| *Posterior* | | 4 | 0.53 | [-5.07, 5.41] |  | 87.1% |
| ***EEG Variable*** | | | | | | |
| ***EEG Measure*** | |  |  |  |  |  |
| *Alpha* | | 5 | 0.51 | [-0.10, 1.16] | 0.55 | 83.90% |
| *SMR* | | 8 | 0.22 | [-0.16, 0.60] |  | 80.60% |
| *Beta* | | 6 | 0.21 | [-0.40, 0.81] |  | 84.50% |

**Supplementary Data. Table S2C. Meta regression**

**Table S2C.** Meta regression of moderators of effects of neurofeedback.

|  | ***Moderator*** | ***k*** | ***β*** | ***95% CI*** | ***p-value*** | ***R^2^*** |
| --- | --- | --- | --- | --- | --- | --- |
| ***First vs. Last NFT*** | |  |  |  |  |  |
|  | *Mean Age* | 24 | -0.01 | [-0.04, 0.02] | 0.57 | 0 |
|  | *Total Training Duration* | 35 | 0.0733 | [-0.01, 0.16] | 0.08 | 6.47% |
| ***First vs. Last NFT (infl. Cases removed)*** | | | |  |  |  |
|  | *Mean Age* | 23 | 0.004 | [-0.02, 0.02] | 0.68 | 0 |
|  | *Total Training Duration* | 34 | 0.0845 | [0.02, 0.15] | 0.02* | 13.19% |
| ***Baseline vs. Post-training*** | | |  |  |  |  |
|  | *Mean Age* | 13 | 0.0036 | [-0.02, 0.02] | 0.68 | 0 |
|  | Total Training Duration | 18 | -0.0637 | [-0.33, 0.21] | 0.62 | 0 |

**References:**

Alexander, F. (2018). Stress coping via musical neurofeedback. *Advances in mind-body medicine*, *32*(2), 17-20.

Anil, K., Demain, S., Burridge, J., Simpson, D., Taylor, J., Cotter, I., et al. (2022). The importance of self-efficacy and negative affect for neurofeedback success for central neuropathic pain after a spinal cord injury. Sci. Rep. 12:10949. doi: 10.1038/s41598-022-15213-7

Cannon, R. L., Baldwin, D. R., Diloreto, D. J., Phillips, S. T., Shaw, T. L., and Levy, J. J. (2014). LORETA neurofeedback in the precuneus: operant conditioning in basic mechanisms of self-regulation. Clin. EEG Neurosci. 45, 238–248. doi: 10.1177/1550059413512796

Cheng, M. Y., Huang, C. J., Chang, Y. K., Koester, D., Schack, T., and Hung, T. M. (2015). Sensorimotor rhythm neurofeedback enhances golf putting performance. J. Sport Exercise Psychol. 37, 626–636. doi: 10.1123/jsep.2015-0166

Christie, S., Bertollo, M., and Werthner, P. (2020). The effect of an integrated neurofeedback and biofeedback training intervention on ice hockey shooting performance. J. Sport Exercise Psychol. 42, 34–47. doi: 10.1123/jsep.2018-0278

de Zambotti, M., Bianchin, M., Magazzini, L., Gnesato, G., and Angrilli, A. (2012). The efficacy of EEG neurofeedback aimed at enhancing sensory-motor rhythm theta ratio in healthy subjects. Exp. Brain Res. 221, 69–74. doi: 10.1007/s00221-012-3148-y

Dekker, M. K., Sitskoorn, M. M., Denissen, A. J., and van Boxtel, G. J. (2014). The time-course of alpha neurofeedback training effects in healthy participants. Biol. Psychol. 95, 70–73. doi: 10.1016/j.biopsycho.2013.11.014

Dempster, T., and Vernon, D. (2009). Identifying indices of learning for alpha neurofeedback training. Appl. Psychophysiol. Biofeedback 34, 309–318. doi: 10.1007/s10484-009-9112-3

Domingos, C., Silva, C. M. D., Antunes, A., Prazeres, P., Esteves, I., and Rosa, A. C. (2021). The influence of an alpha band neurofeedback training in heart rate variability in athletes. Int. J. Environ. Res. Public Health 18:12579. doi: 10.3390/ijerph18231257

Emmert, K., Breimhorst, M., Bauermann, T., Birklein, F., Van De Ville, D., and Haller, S. (2014). Comparison of anterior cingulate vs. insular cortex as targets for real-time fMRI regulation during pain stimulation. Front. Behav. Neurosci. 8:350. doi: 10.3389/fnbeh.2014.00350

Gadea, M., Alino, M., Hidalgo, V., Espert, R., and Salvador, A. (2020). Effects of a single session of SMR neurofeedback training on anxiety and cortisol levels. Neurophysiol. Clin. 50, 167–173. doi: 10.1016/j.neucli.2020.03.001

Gevensleben, H., Albrecht, B., Lütcke, H., Auer, T., Dewiputri, W. I., Schweizer, R., et al. (2014). Neurofeedback of slow cortical potentials: neural mechanisms and feasibility of a placebo-controlled design in healthy adults. Front. Hum. Neurosci. 8:990. doi: 10.3389/fnhum.2014.00990

Göksin, B., Yilmaz, B., and Içöz, K. (2019). Improving short-term memory performance of healthy young males using alpha band neurofeedback. NeuroRegulation 6:15. doi: 10.15540/nr.6.1.15

Guleken, Z., Eskikurt, G., and Karamürsel, S. (2020). Investigation of the effects of transcranial direct current stimulation and neurofeedback by continuous performance test. Neurosci. Lett. 716:134648. doi: 10.1016/j.neulet.2019.134648

Hellrung, L., Dietrich, A., Hollmann, M., Pleger, B., Kalberlah, C., Roggenhofer, E., et al. (2018). Intermittent compared to continuous real-time fMRI neurofeedback boosts control over amygdala activation. Neuroimage 166, 198–208. doi: 10.1016/j.neuroimage.2017.10.031

Hoedlmoser, K., Pecherstorfer, T., Gruber, G., Anderer, P., Doppelmayr, M., Klimesch, W., Schabus, M. (2008). Instrumental conditioning of human sensorimotor rhythm (12–15Hz) and its impact on sleep as well as declarative learning. Sleep 31, 1401–1408.

Keynan, J. N.,Meir-Hasson, Y., Gilam, G., Cohen, A., Jackont, G., Kinreich, S., et al. (2016). Limbic activity modulation guided by functional magnetic resonance imaging- inspired electroencephalography improves implicit emotion regulation. Biol. Psychiatry 80, 490–496. doi: 10.1016/j.biopsych.2015.12.024

Kober, S. E., Neuper, C., and Wood, G. (2020). Differential effects of up-and down-regulation of SMR coherence on EEG activity and memory performance: a neurofeedback training study. Front. Hum. Neurosci. 14:606684. doi: 10.3389/fnhum.2020.606684

Kober, S. E., Witte, M., Ninaus, M., Neuper, C., and Wood, G. (2013). Learning to modulate one’s own brain activity: the effect of spontaneous mental strategies. Front. Hum. Neurosci. 7:695. doi: 10.3389/fnhum.2013.00695

Kober, S. E., Ninaus, M., Witte, M., Buchrieser, F., Grössinger, D., Fischmeister, F. P. S., et al. (2022). Triathletes are experts in self-regulating physical activity but what about self-regulating neural activity?. Biol. Psychol. 173:108406. doi: 10.1016/j.biopsycho.2022.108406

Krogmeier, C., Coventry, B. S., and Mousas, C. (2022). Frontal alpha asymmetry interaction with an experimental story EEG brain-computer interface. Front. Hum. Neurosci. 16:883467. doi: 10.3389/fnhum.2022.883467

Maszczyk, A., Goła´s, A., Pietraszewski, P., Kowalczyk, M., Cieszczyk, P., Kochanowicz, A., and Zajac, A. (2018). Neurofeedback for the enhancement of dynamic balance of judokas. Biol. Sport 35, 99–102. doi: 10.5114/biolsport.2018.71488

Mayeli, A., Misaki, M., Zotev, V., Tsuchiyagaito, A., Al Zoubi, O., Phillips, R., and Bodurka, J. (2020). Self-regulation of ventromedial prefrontal cortex activation using real-time fMRI neurofeedback-Influence of default mode network. Hum. Brain Mapp. 41, 342–352. doi: 10.1002/hbm.24805

Mehran, Y. Z., Firoozabadi, M., and Rostami, R. (2013). Analysis of EEG rhythms under local sinusoidal ELF magnetic field exposure: an approach to neurofeedback enhancement on attention performance. J. Biomed. Sci. Eng. 6, 947–953. doi: 10.4236/jbise.2013.610116

Mirosław, M., and Grzegorz, O. (2022). Neuronal activity in the brain changes during exercise in attention states, warm-up, submaximal effort, and recovery, after neurofeedback-eeg training in motion. Acta Neuropsychol. 20, 175–186. doi: 10.5604/01.3001.0015.8751

Naas, A., Rodrigues, J., Knirsch, J. P., and Sonderegger, A. (2019). Neurofeedback training with a low-priced EEG device leads to faster alpha enhancement but shows no effect on cognitive performance: a single-blind, sham-feedback study. PLoS One 14:e0211668. doi: 10.1371/journal.pone.0211668

Navarro Gil,M., EscolanoMarco, C.,Montero-Marín, J.,Minguez Zafra, J., Shonin, E., and García Campayo, J. (2018). Efficacy of neurofeedback on the increase of mindfulness-related capacities in healthy individuals: a controlled trial. Mindfulness 9, 303–311. doi: 10.1007/s12671-017-0775-1

Patel, K., Henshaw, J., Sutherland, H., Taylor, J. R., Casson, A. J., Lopez-Diaz, K., and Trujillo-Barreto, N. J. (2021). Using EEG alpha states to understand learning during alpha neurofeedback training for chronic pain. Front. Neurosci. 14:620666. doi: 10.3389/fnins.2020.620666

Pérez-Elvira, R., Oltra-Cucarella, J., Carrobles, J. A., Moltó, J., Flórez, M., Parra, S., and Neamtu, B. (2021). Enhancing the effects of neurofeedback training: the motivational value of the reinforcers. Brain Sci. 11:457. doi: 10.3390/brainsci11040457

Pimenta, M. G., van Run, C., de Fockert, J. W., and Gruzelier, J. H. (2018). Neurofeedback of SMR and beta1 frequencies: an investigation of learning indices and frequency-specific effects. Neuroscience 378, 211–224. doi: 10.1016/j.neuroscience.2017.07.056

Rijken, N. H., Soer, R., de Maar, E., Prins, H., Teeuw, W. B., Peuscher, J., and Oosterveld, F. G. (2016). Increasing performance of professional soccer players and elite track and field athletes with peak performance training and biofeedback: a pilot study. Appl. Psychophysiol. Biofeedback 41, 421–430. doi: 10.1007/s10484-016-9344-y

Shibata, K.,Watanabe, T., Kawato, M., and Sasaki, Y. (2016). Differential activation patterns in the same brain region led to opposite emotional states. PLoS Biol. 14:e1002546. doi: 10.1371/journal.pbio.1002546

Staufenbiel, S. M., Brouwer, A. M., Keizer, A. W., and Van Wouwe, N. C. (2014). Effect of beta and gamma neurofeedback on memory and intelligence in the elderly. Biol. Psychol. 95, 74–85. doi: 10.1016/j.biopsycho.2013.05.020

Studer, P., Kratz,O., Gevensleben,H., Rothenberger, A.,Moll, G.H.,Hautzinger,M., et al. (2014). Slow cortical potential and theta/beta neurofeedback training in adults: effects on attentional processes and motor system excitability. Front. Hum. Neurosci. 8:555. doi: 10.3389/fnhum.2014.00555

Tribrat, A. G., Goubkina, D. G., and Pavlenko, V. B. (2007). Dynamics of EEG potentials at the beginning of a series of EEG-feedback sessions. Neurophysiology 39, 82–91. doi: 10.1007/s11062-007-0012-y

van Son, D., van der Does, W., Band, G. P., and Putman, P. (2020). EEG theta/beta ratio neurofeedback training in healthy females. Appl. Psychophysiol. Biofeedback 45, 195–210. doi: 10.1007/s10484-020-09472-1

Weiss, F., Zhang, J., Aslan, A., Kirsch, P., and Gerchen, M. F. (2022). Feasibility of training the dorsolateral prefrontal-striatal network by real-time fMRI neurofeedback. Sci. Rep. 12:1669. doi: 10.1038/s41598-022-05675-0

Zhang, G., Zhang, H., Li, X., Zhao, X., Yao, L., and Long, Z. (2012). Functional alteration of the DMN by learned regulation of the PCC using real-time fMRI. IEEE Trans. Neural Syst. Rehabil. Eng. 21, 595–606. doi: 10.1109/TNSRE.2012.2221480
