# Supplementary figures and images for: Systematic review and meta-analysis of the relationships between real-time neurofeedback training parameters and acquisition of neural modulation
Source: Front Hum Neurosci. 2025 Aug 29;19:1652607. doi: 10.3389/fnhum.2025.1652607 (PMC12426165; doi:10.3389/fnhum.2025.1652607)

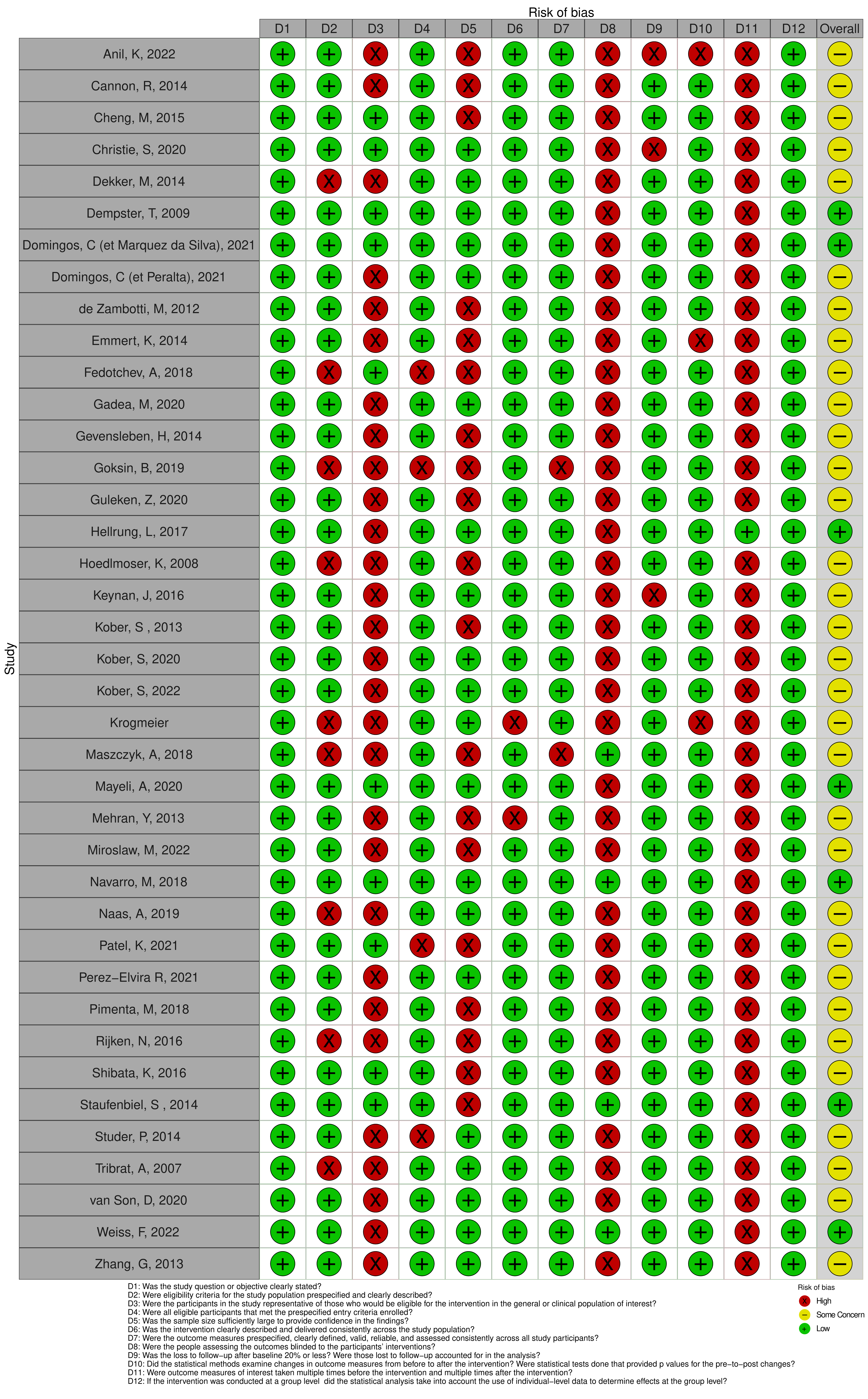

Supplement: Supplementary file 4 [file Image_1.png]
